# Spike-Representation of EEG Signals for Performance Enhancement of Brain-Computer Interfaces

**DOI:** 10.3389/fnins.2022.792318

**Published:** 2022-04-04

**Authors:** Sai Kalyan Ranga Singanamalla, Chin-Teng Lin

**Affiliations:** ^1^Computational Intelligence and Brain Computer Interface Lab, School of Computer Science, University of Technology Sydney, Sydney, NSW, Australia; ^2^Australian Artificial Intelligence Institute, University of Technology Sydney, Sydney, NSW, Australia

**Keywords:** spiking neural network, brain-computer interface, electroencephalography, P300, error-related negativity, classification

## Abstract

Brain-computer interfaces (BCI) relying on electroencephalography (EEG) based neuroimaging mode has shown prospects for real-world usage due to its portability and optional selectivity of fewer channels for compactness. However, noise and artifacts often limit the capacity of BCI systems especially for event-related potentials such as P300 and error-related negativity (ERN), whose biomarkers are present in short time segments at the time-series level. Contrary to EEG, invasive recording is less prone to noise but requires a tedious surgical procedure. But EEG signal is the result of aggregation of neuronal spiking information underneath the scalp surface and transforming the relevant BCI task's EEG signal to spike representation could potentially help improve the BCI performance. In this study, we designed an approach using a spiking neural network (SNN) which is trained using surrogate-gradient descent to generate task-related multi-channel EEG template signals of all classes. The trained model is in turn leveraged to obtain the latent spike representation for each EEG sample. Comparing the classification performance of EEG signal and its spike-representation, the proposed approach enhanced the performance of ERN dataset from 79.22 to 82.27% with naive bayes and for P300 dataset, the accuracy was improved from 67.73 to 69.87% using xGboost. In addition, principal component analysis and correlation metrics were evaluated on both EEG signals and their spike-representation to identify the reason for such improvement.

## 1. Introduction

Using the brain signals to communicate with external devices without the intervention of explicit human motor actions, called Brain-Computer Interfaces (BCI), provides a new dimension to interactive technology (Allison et al., 2007; Rashid et al., 2020). Notable examples of BCI application include controlling of wheelchair (Zhang et al., 2015), exoskeleton (Wang et al., 2018), speller (Rezeika et al., 2018), gaming (Ahn et al., 2014), etc. Apart from commercial prospects, BCIs have also been proved to be efficient in various clinical applications, for example, by-passing information from the brain to hand muscle through external stimulation due to the damage of motor nerves (Pohlmeyer et al., 2009), restoration of lost functionality with BCI training (Grosse-Wentrup et al., 2011), etc. As such, BCI has shown tremendous possibility toward its integration into daily life activities and clinical rehabilitation. The most common mode of neuroimaging method utilized for brain signal assessment is Electroencephalography (EEG) due to its high temporal resolution and ease of usage (Min et al., 2010; Zhang et al., 2010). Rapid developments in EEG acquisition devices enabled it to be user-friendly, portable and transit data wirelessly. Depending on the application, different number of EEG channels and modalities such as Motor imagery (MI) (Padfield et al., 2019), Steady-state Visually Evoked Potentials (SSVEP) (Bin et al., 2009) and Event-related potentials (ERP) (Kaufmann et al., 2011; Mayaud et al., 2013). In parallel, advances have been made toward bridging the gap between laboratory settings and real-world usage by enhancing EEG-based BCI systems performance. To this end, studies have focused on the development of novel machine learning methods and feature extraction techniques to improve the BCI system performance. In particular, Deep learning studies have shown promising results in this regard (Manor and Geva, 2015; Lawhern et al., 2018). For example, Lawhern et al. (2018) introduced EEGNet based on convolution neural network and has shown improved performance on various BCI modalities. However, few shortcomings need to be accounted for to yield complete advantage of such a deep learning model such as the availability of a large sample of training data, more EEG channels, fine-tuning model parameters, etc. For example, Lawhern et al. (2018) used approximately 2,000 samples for P300 classification, more than 6,000 samples by Manor and Geva (2015) for RSVP classification, and more than 10,000 samples by Ma et al. (2021). In practice, EEG data collections require a tedious experimental setup and are often time-consuming for a user to record huge datasets. Alternatively, classical techniques though can execute with fewer data samples but lack efficient feature representation. However, EEG signals recorded over the scalp surface are the collective information of multiple neuronal action potentials underneath the surface. We hypothesize that extracting spiking information from EEG signals could potentially help advance the BCI performance.

A model that naturally accommodates spiking information is Spiking Neural Network (SNN) and it is the third generation of neural networks. Contrary to artificial neural networks, they communicate *via* spikes mimicking the properties of the biological neural circuit, and as such SNNs have been gaining traction in the field of machine learning (Bellec et al., 2018; Woźniak et al., 2020). For example, Bellec et al. (2018) has leveraged adaptive properties of spiking neurons to obtain Long-Short-Term-Memory (LSTM) equivalent performance. Similarly, few studies have shown the efficiency of SNN toward speech recognition (Wu et al., 2020), objection detection (Kim et al., 2020), and language modeling (Woźniak et al., 2020). Also, SNN was examined in its ability to classify non-stationary EEG signals. For example, Antelis et al. (2020) encoded Motor Imagery (MI)-based EEG to spiking data for assessing the classification performance by SNN. Apart from BCI application, few studies have shown that SNN can also be adopted as classifiers for emotion recognition (Luo et al., 2020), seizure detection (Ghosh-Dastidar and Adeli, 2009), understand functional connectivity changes due to mind-fullness training (Doborjeh et al., 2019), and depression (Shah et al., 2019). These mentioned studies assume SNN to be a classifier as a standard notion similar to artificial neural networks, where the input (such as EEG) is provided to the network and it outputs a class label.

Recently, besides classification, our previous study has shown that SNN can be adopted as a generator model to efficiently generate artificial EEG signals to tackle the data augmentation problem of MI-based BCI systems and improve the performance (Singanamalla and Lin, 2021). In continuation of our previous work, we believe that reverse engineering SNN to extract spiking information from EEG signals could also improve BCI system performance. Similar to our previous work, the SNN is trained to produce task-relevant multi-channel EEG template signals. However, a major difference in this study is that, instead of using a trained model to create artificial samples, the model is used to extract spike-representation of EEG sample and this spike-representation is evaluated against baseline EEG classification performance. Major bottleneck issues for the practical outdoor usage of the BCI system are the noise/artifacts, fewer channels (for compactness), and fewer samples (less training time). Therefore for this study, two different BCI modalities, P300 and Error-Related Negativity (ERN) were primarily focused as they contain their biomarker in the time-series and as such more sensitive to noise. In addition, considering the above bottleneck issues, datasets with limited channels and fewer samples were considered to evaluate the proposed approach. Based on the result, we have observed that the proposed approach could potentially improve the BCI performance. This study further explored the reasoning as to why there is a performance enhancement due to the spike representation.

## 2. Methods

### 2.1. Neuron Model

Each node in an SNN adheres to the dynamics of a spiking neuron and computes on a time-scale contrary to an artificial neuron which acts as a non-linear function. A family of spiking neuron models offering different levels of detailed simulation & complexity exists such as Hodgkin Huxley (HH) model (Hodgkin and Huxley, 1952), Izhikevich model (Izhikevich, 2003), Spike-Response model (SRM) (Jolivet et al., 2003), Leaky-Integrate and Fire (LIF) model (Neftci et al., 2019), Spiking Neural Unit (SNU) (Woźniak et al., 2020) etc. LIF model is the widely adopted model for building SNN due to its computational simplicity and efficiency. The membrane potential dynamics of a LIF neuron is described according to Equation (1).


(1) $$\begin{array}{r} \;\tau_{\text{m}}\frac{\text{d}u_{i}}{\text{d}t}=-\left(u_{i}-u_{\text{rest}}\right)+RI_{i} \end{array}$$

where *u*_*i*_(*t*) describes the membrane potential of the neuron indexed by *i* and it's input current is presented by *I*_*i*_(*t*) at a given time point *t*. The constant parameters of this model include, baseline resting membrane potential *u*_*rest*_, membrane time constant τ_*m*_ , resistance *R*, and the synaptic time constant τ_*s*_. The input current dynamics is described by Equation (2).


(2) $$\begin{array}{r} \frac{\text{d}I_{i}}{\text{d}t}=-\frac{I_{i}\left(t\right)}{\tau_{\text{s}}}+\underset{j}{\sum }W_{ij}S_{j}\left(t\right) \end{array}$$

(3) $$\begin{array}{r} \;S_{i}\left(t\right)=\underset{k}{\sum }\delta \left(t-t_{i}^{k}\right) \end{array}$$

*S*_*i*_(*t*) is the spike train (sum of a dirac delta function, δ) of a neuron and $t_{i}^{k}$ is the *k*^*th*^ firing time of the corresponding neuron. The membrane potential increases with input current, and after reaching a threshold ϑ, the neuron emits a spike and resets its potential to *u*_*rest*_. By incorporating this reset property the updated membrane dynamics is presented by Equation (4):


(4) $$\begin{array}{r} \;\frac{\text{d}u_{i}}{\text{d}t}=-\frac{1}{\tau_{\text{m}}}\left(u_{i}-u_{\text{rest}}\right)+RI_{i}+S_{i}\left(t\right)\left(u_{\text{rest}}-\vartheta \right) \end{array}$$

For ease of use, the membrane dynamics can be further simplified, as suggested in Neftci et al. (2019), where an approximated version of the LIF membrane potential and input current for a small simulation time step Δ_*t*_>0, as shown in Equations (5, 6), by incorporating the numerical value of *R*, *u*_*rest*_, and ϑ from Table 1.


(5) $$\begin{array}{r} I_{i}\left[n+1\right]=\alpha I_{i}\left[n\right]+\underset{j}{\sum }W_{ij}S_{j}\left[n\right] \end{array}$$

(6) $$\begin{array}{r} u_{i}\left[n+1\right]=\beta u_{i}\left[n\right]+I_{i}\left[n\right]-S_{i}\left[n\right] \end{array}$$

where $\alpha \equiv \exp\left(-\frac{\Delta_{t}}{\tau_{\text{s}}}\right)$, $\beta \equiv \exp\left(-\frac{\Delta_{t}}{\tau_{\text{m}}}\right)$.

**Table 1:** List of spiking neuron and Adam optimizer properties.

	**Value**
**Spiking neuron**	
*dt*	1 mS
*u*_*rest*_	0 mV
τ_*m*_	10 mS
τ_*s*_	5 mS
*R*	1 M
ϑ	1 mV
τ_*d*_	2 mS
τ_*r*_	3 mS
*l*(τ)	0.8
**Optimizer**	
Learning rate	0.0005
Betas	(0.9, 0.999)
Epsilon	8 × 10^−1^
Weight decay	0

In addition to LIF, we have also tested SNU (whose dynamics are represented by Equation 7) based SNN model. The advantage of SNU is that it can act as both artificial and spiking neuron depending on the choices of activation function. To compare with LIF, here we choose activation functions *g* = *relu* and *h* = *heaviside* to implement spiking functionality.


(7) $$\begin{array}{r} \begin{array}{c} {\text{u}}_{t}=g\left(W{\text{x}}_{t}+l\left(\tau \right)⊙{\text{u}}_{t-1}⊙\left(1-{\text{s}}_{t-1}\right)\right)\\ {\text{s}}_{t}=h\left({\text{u}}_{t}-\vartheta \right) \end{array} \end{array}$$

where, *x*_*t*_, *u*_*t*_, *s*_*t*_ represent the input, membrane potential and spike output, respectively at time step *t*. A detailed list of all the parameter of LIF and SNU are presented in Table 1.

### 2.2. SNN Architecture

**Figure 1:**
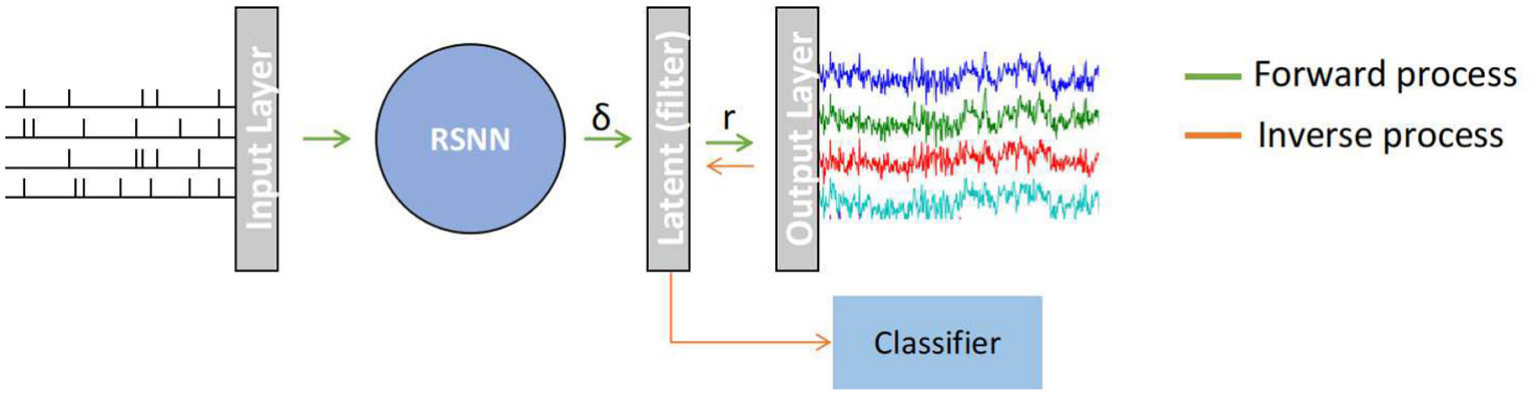
A recurrent spiking neural network (RSNN) model that maps a random input spike-train from *Input Layer* to a multi-channel EEG signal in the *Output Layer*. The spike trains (δ) from RSNN are processed in the latent layer to continuous signal *r* using a double exponential synaptic filer before converting to an EEG signal. This model works in two stages: (1) Forward process in which SNN generates template EEG signal, (2) Inverse process in which the non-template signal are transformed to spike-representation for classification.

A recurrent based SNN model (RSNN) in which each node is based on a simplified LIF or SNU model and computes following Equation (5) for producing EEG signals. Similar to Singanamalla and Lin (2021), since the source spiking information that generates desired EEG signal is often not available, the input layers generate spike-trains adhering to Poisson distribution with a firing rate of 10 Hz. This spike train is then processed *via* the RSNN population. Since EEG signal is continuous contrary to spike-trains (discrete-time series), a double exponential synaptic filter (see Section 2.2.2) is applied to the neural activity of RSNN to smoothen the spike-trains, denoted as a latent layer. The filtered spike-trains are then weighted averaged to produce a multi-channel EEG signal. An overview of the proposed architecture is depicted in Figure 1. The proposed architecture comprises a different number of nodes(or neurons) in each layer as follows: 50 for the input layer, 100 for RSNN, 100 for the latent layer, and 2 for the output layer. The model was built using *pytorch*, a popular python-based deep learning library, and the weight connections between the all layers and recurrent weights within RSNN are trained using surrogate-gradient descent (see Section 2.2.1) with *Adam* optimizer.

In a given EEG dataset, no two samples are the same for a defined stimulus. So, training the model for each EEG sample independently to obtain latent information (i.e., the spike-representation of EEG sample) is computationally demanding. Therefore, a representative EEG signal, called template EEG, was initially obtained from a given dataset for the training process. As suggested in Singanamalla and Lin (2021), a classifier was trained and tested with a dataset and the sample with the highest probability of being categorized correctly into its respective class will be considered as the EEG template.

The model is implemented in a two-stage process namely, *Forward process* and *Inverse process*. In the *Forward process*, the model was trained using surrogate gradient descent to reproduce multi-channel EEG templates of a given dataset. Following this, the *Inverse process* transforms each of the non-template signals (i.e., samples other than template in a given dataset) to its spike-representation by Equation (8) in which the pseudo-inverse of trained weights (connecting latent-layer to output layer) is multiplied by EEG samples.


(8) $$\begin{array}{r} r_{j}=W_{f}^{-1}*EEG_{j} \end{array}$$

where *W*_*f*_ is the trained weight matrix from latent layer to EEG signal output.

#### 2.2.1. Surrogate-Gradient Descent

Gradient descent optimization is the most widely adopted optimization technique in an artificial neural network. Gradient estimation requires the function to be continuous such that it is differentiable. However, for a spiking neuron's spike emission process mimics the step activation function, i.e., when the voltage exceeded its threshold a spike is produced and resets immediately. As such, the resulting discontinuous function limits the usage of the standard gradient descent algorithm. To this end, Neftci et al. (2019) developed an approach called surrogate-gradient descent, in which the step activation applied to membrane voltage at a time step *t*, *S*_*i*_[*t*]∝Θ(*u*_*i*_[*t*]−ϑ), during forward propagation is replaced with *Sigmoid* activation function during backpropagation as follows: σ(*u*_*i*_[*t*]−ϑ), where σ(*x*) = 1/(1+*exp*(−*x*)). This flexibility of using pseudo-derivatives during backward propagation enables the use of gradient descent optimization variants. It is important to note that, the sigmoid replacement happens only during backpropagation while the step function remains fixed during forward propagation.

#### 2.2.2. Synaptic Filter

A spike train is a discontinuous time series with a value of 1 (spike) or 0 (no-spike) at each time step. Therefore, as suggested in Nicola and Clopath (2017), a double exponential synaptic filter is applied (latent layer) to the spike trains of RSNN in the *Forward process* to obtain a continuous signal. This filtered signal is later transformed into a multi-channel (2 channels in this study) EEG signal. For a spike train from one node, the synaptic filter is estimated according to Equation (9).


(9) $$\begin{array}{r} \begin{array}{c} {ṙ}_{j}=-\frac{r_{j}}{\tau_{d}}+h_{j}\\ {ḣ}_{j}=-\frac{h_{j}}{\tau_{r}}+\frac{1}{\tau_{r}\tau_{d}}\sum_{t_{j}<t}\delta \left(t-t_{jk}\right) \end{array} \end{array}$$

where, τ_*r*_ represents synaptic rise time, τ_*d*_ is the synaptic decay time, and δ is the spike train of *RSNN* layer (see Figure 1). The spike is first filtered with one intermediary exponential filter *h*_*j*_ and followed by another exponential filter *r*_*j*_. The activity of *r*_*j*_ is the output of *latent* layer.

### 2.3. EEG Processing

Two types of ERP signal, Error-Related negativity (ERN) and P300, are considered for this study. The datasets are publicly available from Van Veen et al. (2019) and Kappenman et al. (2021), respectively. A standard EEG processing pipeline containing bandpass filtering between 0.5 and 45 Hz, epoch extraction, and channel selection were applied to each of the datasets. For both ERN and P300 datasets, 20 subject data were considered. A template EEG signal was extracted (as mentioned in Section 2.2) for each subject dataset independently and smoothed using a moving average window. This template was later utilized for training the SNN model. For SNU-based model, the signal is also normalized from −1 to 1 for better fitting.

### 2.4. Classification

For ERN and P300 signals, the EEG time series data are considered as features for classification. The most predominantly used classifiers for both ERN and P300 include Support Vector Machine's (SVM), Gaussian classifiers, and tree-based algorithms (Ventouras et al., 2011; Chavarriaga et al., 2014; Sarraf et al., 2021). Therefore, this study had adopted these classifiers for assessing the baseline EEG classification accuracy. For a fair comparison, the spike-representation (with both LIF and SNU-based SNN) of EEG signals was classified with the same classifiers. A 5 × 10-fold cross-validation (CV) was applied for each subject data and the overall performance of given subject data is the average of all the folds. To test the significance in performance improvement with spike-representation, a two-sided wilcoxon signed-rank test was performed on the classification accuracies obtained from each classifier independently. Refer to Section 3.2 on the outcomes of classification and significance test results.

### 2.5. Explainability

Based on the classification results (see Section 3.2), we observed that the performance be enhanced above baseline by transforming the EEG signals to spike-representation. Debugging the reasoning behind such accuracy increment could better help understand the neural origins of EEG signals and to why EEG signal is often ineffective compared to invasive electrode recordings. Principal component analysis (PCA) was applied to the EEG data and its corresponding spike-representation to estimate the variance explanation ratio. This assisted in exploring if the proposed approach was able to extract novel features (or more independent variables). In addition, we have compared the confusion matrix analysis and correlation properties to explore the attributes of spike-transformed data.

## 3. Results

### 3.1. SNN Training

**Figure 2:**
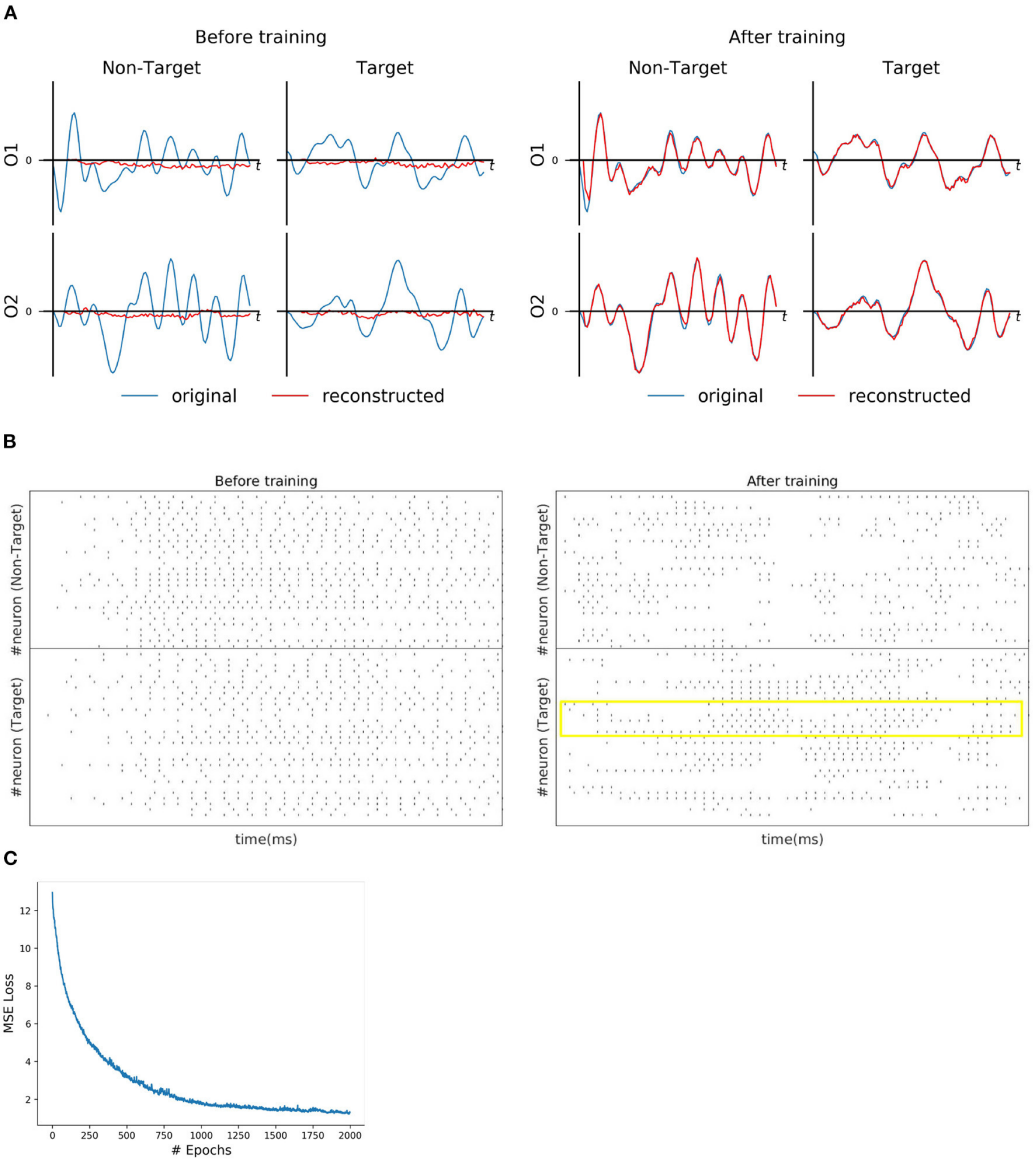
P300 signal reconstruction outcomes. **(A)** Illustration of original (blue line) and reconstructed (red line) for O1 and O2 EEG signals for both *Target* and *Non-Target* classes before and after training. **(B)** Spike trains of RSNN activity before and after the training process from 30 randomly selected neurons. The yellow box indicates the effectiveness of the training process in reorganizing the spike trains across layers. **(C)** The convergence of MSE loss value across epochs during the training process.

**Figure 3:**
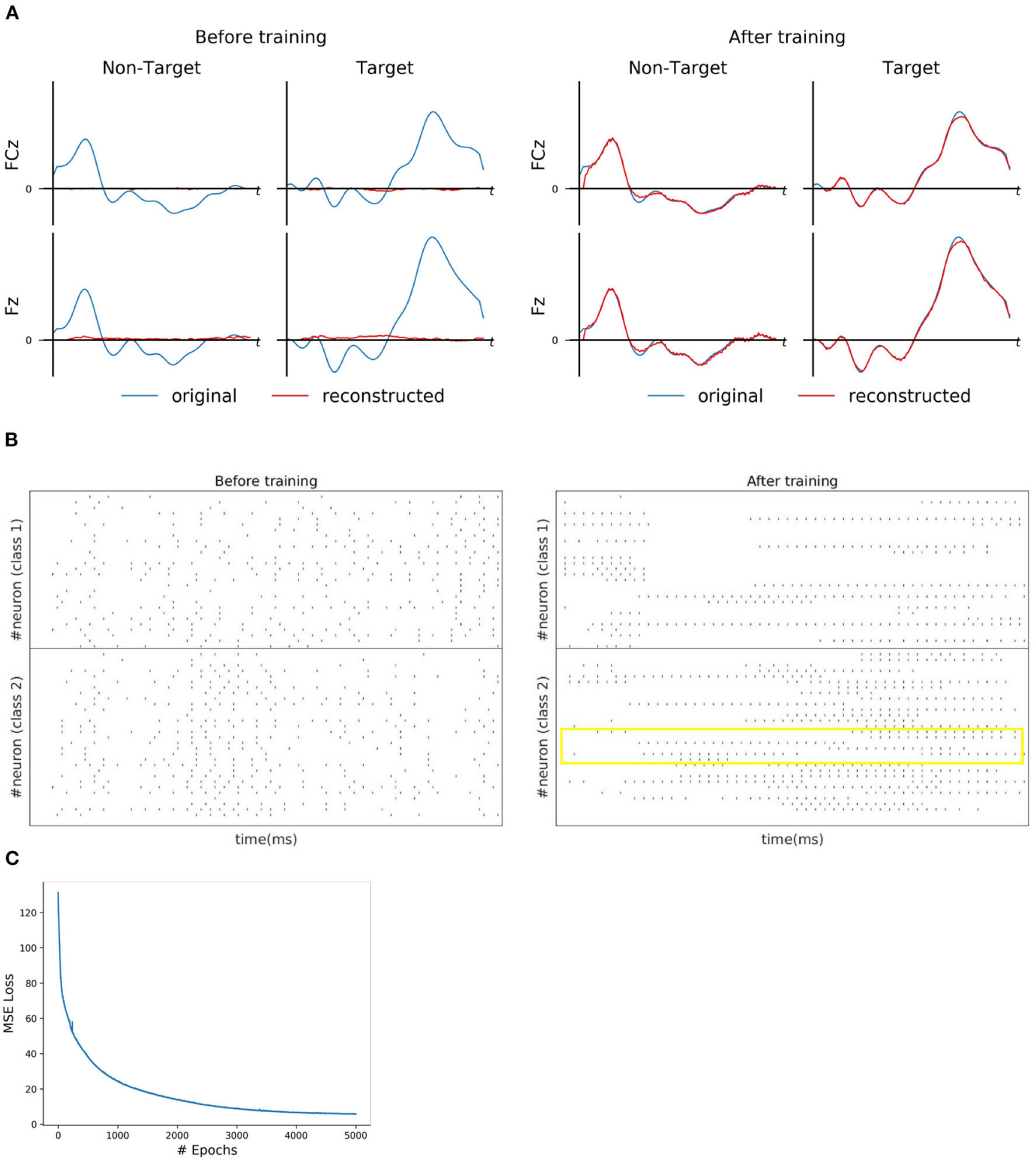
ERN signal reconstruction outcomes. **(A)** Illustration of original (blue line) and reconstructed (red line) for FCz and Fz EEG signals for both *Target* and *Non-Target* classes before and after training. **(B)** Spike trains of RSNN activity before and after the training process from 30 randomly selected neurons. The yellow box indicates the effectiveness of the training process in reorganizing the spike trains across layers. **(C)** The convergence of MSE loss value across epochs during the training process.

For each subject dataset, a new instance of the model (LIF or SNU-based SNN) was trained to generate a set of EEG template signal. In the case of ERN and P300 datasets, this set comprises of two template signals (one from each class denoted as target and non-target). Accordingly, two Poisson spike trains (at 10 Hz rate) were initialized to acts as input signal to the SNN model. For some subjects as shown in Figures 2A, 3A, the model was able to reproduce these template EEG signals for both P300 and ERN signals, respectively. Also, it can be observed that RSNN activity was rearranged during the training process for both ERN (see Figure 2B) and P300 EEG signal (see Figure 3B). Since the objective of the model is to reproduce a time series signal (i.e., EEG template), a Mean Square Error (MSE) loss function was adopted as a cost function. From Figures 2C, 3C, it can be observed this loss converged quickly for both P300 and ERN EEG signal generation. A detailed list of neuron and optimizer parameter are provided in Table 1.

### 3.2. Classification

#### 3.2.1. P300

In the P300 analysis, 20 subjects' data from open source (Van Veen et al., 2019) was considered for this study. The datasets were pre-processed according to the pipeline mentioned in Section 2.3. As previously mentioned the limits of practicality, only two channels that contribute to P300 bio-maker, FCz & Fz were considered toward the further process. To extract the template signal from a given subject dataset, all the samples in the dataset are used for both training and testing with the SVM classifier. The sample that obtained the highest probability of being correctly categorized into its class is considered as the template. Since P300 is a binary class (Target and Non-target) system, we obtained two EEG templates, one for each class. After training the model to generate this EEG template, each of the remaining samples in the data (i.e., non-template) were converted to its spike representation through Equation (8). Then, different classifiers such as SVM, Naive Bayes (NVB), and xGboost were used for comparing the performance of EEG signals (baseline performance) and their respective spike-representation signals. For each subject, classification accuracy represents the average of a 5 × 10-fold CV (i.e., 5 *times* 10-fold), in which each fold was executed with 90%of the randomly selected samples as training set and remaining for the testing set. As shown in Table 2, using xGboost, spike-representation of EEG signals has obtained an average accuracy of 69.87% (with SNU-based SNN), 69.56% (with LIF-based SNN), and exceeded the baseline performance (EEG signal classification accuracy) of 67.73%. In addition, few subjects (such as S13 and S19) showcased significant improvement from baseline performance by up to 5–7%. A one-sided wilcoxon-signed rank test was applied between accuracies obtained from EEG and their spike-representation (either from LIF or SNU) from each classifier separately. This statistical significance analysis resulted in a *p* < 0.05 for all the classifiers.

**Table 2:** Comparison of classification performance of P300 based EEG signal and its spike-representation by LIF-based and SNU-based SNN using SVM, NVB, and xGboost classifiers.

**Dataset**	**SVM**			**NVB**			**xGboost**		

	**EEG**	**LIF**	**SNU**	**EEG**	**LIF**	**SNU**	**EEG**	**LIF**	**SNU**
S1	69.39	75.39	72.82	76.03	77.52	77.38	71.02	71.61	71.61
S2	69.70	70.63	71.42	71.36	72.78	72.62	68.47	68.05	68.07
S3	66.69	70.91	70.82	69.42	70.36	72.54	67.99	71.37	71.53
S4	72.94	77.96	76.23	82.18	84.20	83.11	83.09	83.27	83.59
S5	58.66	62.96	62.65	62.78	63.24	63.73	61.24	65.04	64.10
S6	70.50	74.89	75.05	66.31	71.58	71.74	69.57	71.91	72.49
S7	61.14	67.80	67.49	67.16	67.70	67.15	66.88	66.19	66.91
S8	70.50	74.81	74.19	66.31	71.42	71.58	69.57	71.91	72.90
S9	67.54	69.29	68.98	70.32	69.99	68.65	72.60	71.89	71.89
S10	57.33	58.59	58.27	58.91	61.22	63.52	59.63	58.35	62.00
S11	62.29	63.75	62.96	58.38	63.40	65.75	65.62	66.41	66.47
S12	81.69	82.95	82.78	76.26	83.21	86.27	84.86	86.23	85.53
S13	57.90	60.01	60.32	60.43	60.96	63.24	55.88	62.66	62.26
S14	54.77	57.01	56.77	56.98	57.99	57.91	57.83	59.66	59.48
S15	62.82	63.40	63.96	64.28	66.79	67.82	63.94	62.71	62.54
S16	64.18	69.81	70.35	66.57	71.16	71.55	67.22	69.70	71.90
S17	67.16	68.01	68.41	64.99	65.33	66.28	70.84	74.06	74.44
S18	60.95	68.26	68.50	65.00	66.83	69.93	64.60	68.95	68.88
S19	69.89	69.18	69.50	72.44	71.50	70.02	68.65	73.70	73.94
S20	59.24	63.41	64.24	64.58	63.20	62.26	65.08	67.48	66.78
**Avg**	65.26	68.45	68.29	67.04	69.02	**69.65**	67.73	69.56	**69.87**

*For each subject, a 5 × 10 CV fold was applied where 90% of the samples were randomly selected for training and the remaining 10% for testing in each fold. The bold values indicate the highest performance produced from all models*.

#### 3.2.2. ERN

The SNN model training process for ERN was similar to P300 except that the channels selected for this dataset are Pz & Oz. In total 20 subject datasets were considered for assessing the spike-representation competency for this BCI mode. Similar to the P300 assessment in Section 3.2.1, the template signal for each subject was extracted based on the probability (obtained by SVM) of each sample to be categorized into its respective class. The classification performance of the ERN signal and its spike representation was evaluated using SVM, NVB, and xGboost classifiers. As shown in Table 3, with NVB, spike-representation of EEG signals has obtained an accuracy of 82.27% (with LIF-based SNN), 81.66% (with SNU-based SNN) and exceeded the baseline (EEG) performance of 79.22%. In addition, few subjects (such as S15) have shown a significant increase (up to 7%) with spike-representation. Similar to P300, the statistical significance analysis for ERN using one-sided wilcoxon signed-rank test has also resulted in a *p* < 0.05 for all the classifiers with LIF and for SNU *p*-values are 0.082 (for SVM), 0.018 (for SVB), and 0.095 (for xGboost).

**Table 3:** Comparison of the classification performance of ERN's EEG signal and its spike-representation by LIF-based and SNU-based SNN using SVM, NVB, and xGboost classifiers.

**Dataset**	**SVM**			**NVB**			**xGboost**		

	**EEG**	**LIF**	**SNU**	**EEG**	**LIF**	**SNU**	**EEG**	**LIF**	**SNU**
S1	69.45	67.56	69.09	77.73	80.21	79.86	74.44	74.27	73.17
S2	92.83	93.00	92.33	91.83	91.83	90.50	80.67	82.00	85.00
S3	77.25	76.39	76.97	75.00	78.44	78.00	70.92	81.69	78.83
S4	86.52	86.12	85.54	88.73	89.44	89.57	86.05	89.51	89.67
S5	70.22	72.02	70.87	76.15	76.95	76.96	79.06	75.10	75.08
S6	81.67	86.33	85.00	91.67	93.67	93.00	69.67	73.33	73.33
S7	81.51	82.80	82.82	72.60	76.18	74.22	78.98	85.36	84.31
S8	69.30	72.78	70.84	63.78	58.53	58.65	64.68	74.23	72.91
S9	78.00	76.52	78.19	80.43	83.76	82.57	78.81	78.81	80.67
S10	87.29	86.73	87.43	88.27	90.52	90.79	90.41	89.61	89.08
S11	77.87	80.00	80.13	84.00	86.00	86.27	85.60	83.07	82.80
S12	67.00	73.67	74.33	76.33	75.33	70.33	53.33	80.67	80.33
S13	87.60	88.76	88.96	90.96	91.36	92.73	90.96	88.78	89.56
S14	81.09	81.71	80.60	87.56	89.02	89.24	90.64	87.80	88.51
S15	84.30	91.30	91.70	83.10	90.60	89.70	80.80	89.60	91.00
S16	55.00	61.00	80.67	59.00	63.67	71.73	63.67	60.67	80.97
S17	77.00	80.00	60.00	73.00	95.00	63.00	90.00	90.00	58.67
S18	79.87	79.60	80.00	79.76	82.02	94.00	81.60	79.60	70.00
S19	71.38	72.77	79.47	76.77	78.62	82.58	78.15	82.92	78.64
S20	80.04	85.63	72.31	67.81	74.27	79.54	74.32	80.83	81.54
**Avg**	77.76	79.73	79.36	79.22	**82.27**	81.66	78.14	81.39	80.20

*For each subject, a 5 × 10 CV fold was applied where 90% of the samples were randomly selected for training and the remaining 10% for testing in each fold. The bold values indicate the highest performance produced from all models*.

To further assess the impact of SNN on performance improvement, we have transformed the ERN signal to a high-dimension signal (in Equation 8) in two ways as a control study: (1) using a random weight matrix (of normal distribution equivalent to initialized SNN weights) and (2) Liquid State Machines (LSM; based on LIF) in which all RSNN related weight matrices (feedforward & recurrent) are fixed except for the output layer weights that convert filtered signal *r* to EEG signal during the model training process. The performance of the transformed signal with these two approaches is estimated using NVB as it has obtained the best performance previously. As shown in Table 4, the average performances with random weight is 80.70% (*p* = 0.287 with one-sided wilcoxon test) and LSM is 81.83% with *p* = 0.74. The effect of SNN's trained weights on performance is not significantly higher than these control methods, but provided an additional edge in the average performance.

**Table 4:** Comparison of classification performance of ERN dataset *via* NVB for transformed signal with random weight and LSM.

**Dataset**	**Random-W**	**LSM**
S1	79.35	82.12
S2	91.83	89.17
S3	77.56	77.25
S4	89.86	88.71
S5	76.81	76.81
S6	93.00	93.67
S7	74.36	76.40
S8	61.56	59.20
S9	82.00	81.24
S10	89.33	91.88
S11	85.60	86.27
S12	70.33	72.33
S13	91.33	92.15
S14	87.96	90.29
S15	90.50	89.70
S16	70.79	72.38
S17	60.00	62.00
S18	85.00	94.00
S19	82.02	82.22
S20	76.62	78.92
**Avg**	80.79	81.84

### 3.3. Confusion Matrix

**Figure 4:**
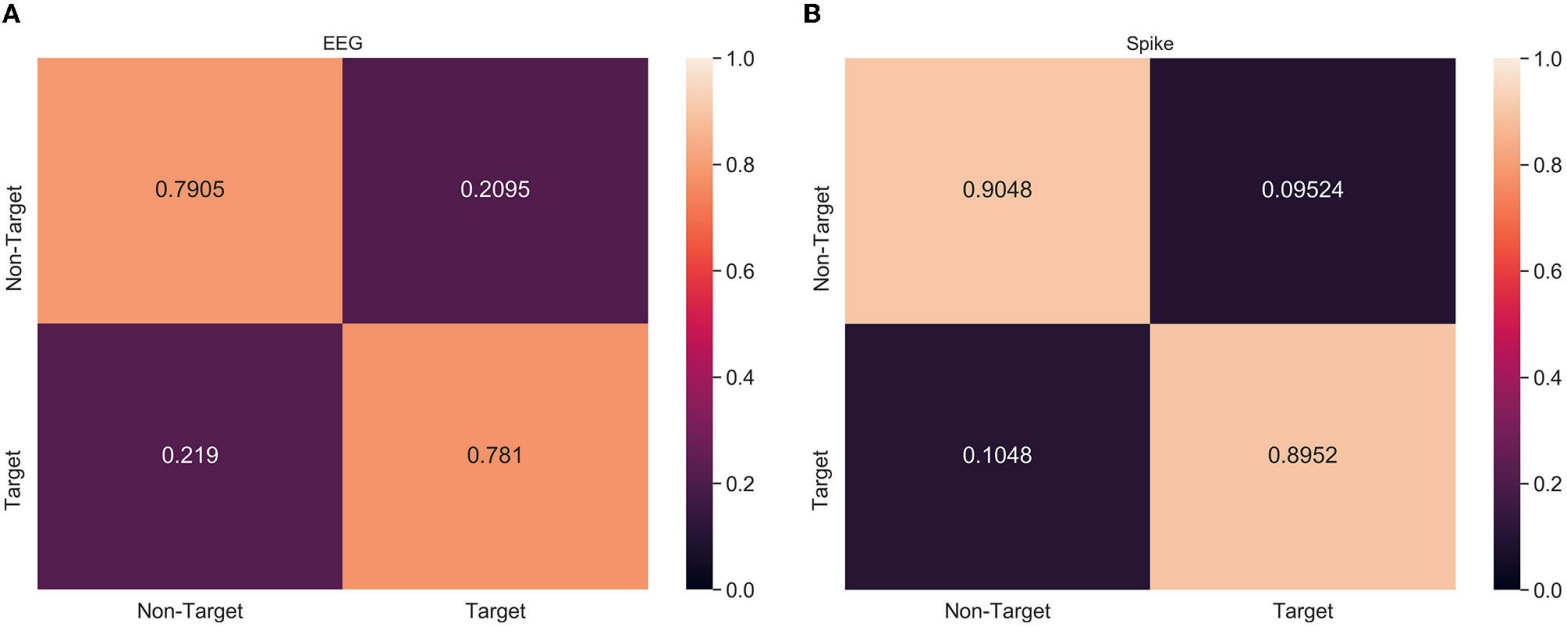
Confusion matrix analysis of **(A)** EEG and **(B)** Spike-representation (from LIF) of ERN dataset. Recognition of both *Target* and *Non-Target* categories improved for spike-representation compared to EEG signal.

From Tables 2, 3, it can be observed that spike-representation can improve the classification performance. However, the classification metric alone does not provide enough details on the model performance. Therefore, confusion matrix analysis with xGboost classifier was performed on a select subject, from both ERN and P300 datasets, that had significant improvement over baseline performance. Based on Figure 4, we found that for ERN, the proposed approach increased both true positive and false negative. This indicates that the spike representation was able to account for patterns of both target and non-target samples and improve their detection rate. Similar behavior was identified for the P300 dataset as well (see Supplementary Figure 1).

### 3.4. Explainability

Though EEG signal and its spike-representation are directly analogous, spike-representation of the data was able to provide better performance. Exploring the reason behind the performance increment could better help in further enhancing the credibility of BCI systems. To this end, PCA and correlation measures were applied to both EEG signal and its spike-representation data to assess the internal factor that leads to performance enhancement.

#### 3.4.1. Variance Explanation Analysis

**Figure 5:**
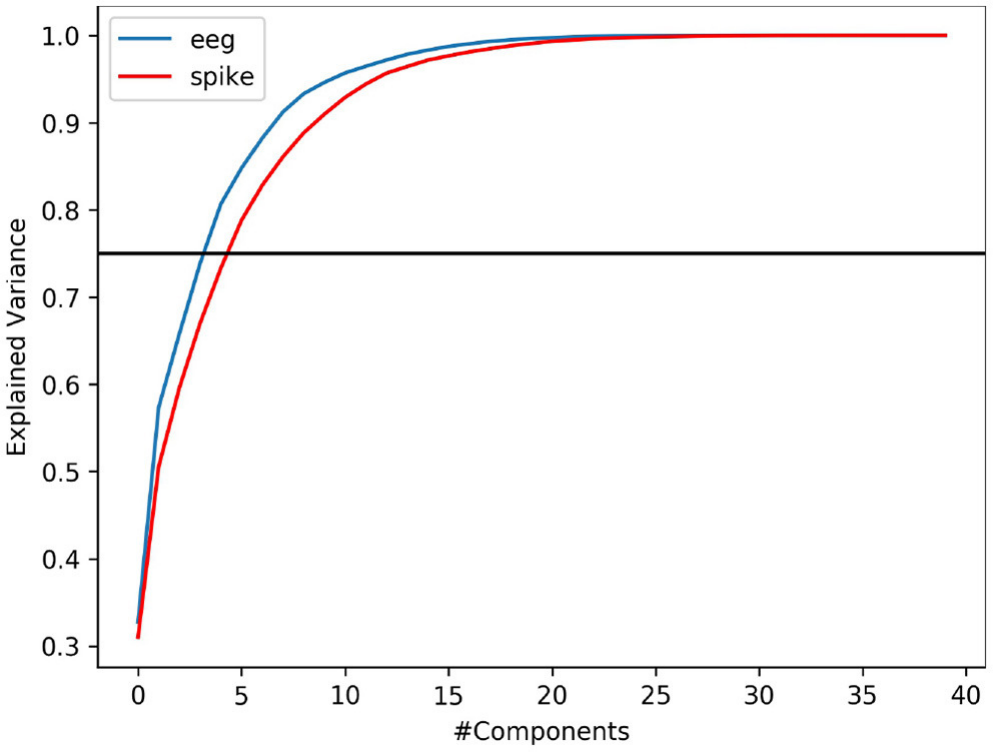
PCA-based variance explanation of EEG signal (blue line) and its corresponding spike representation (from LIF; red line) for ERN dataset. Blackline represents the 75% variance. Spike-representation (from LIF) was able to extract a more independent eigenvector from EEG signal to account for a given fraction of variance in the dataset.

It is common that not all variables in a data is useful to provide meaningful information. Explained variance analysis is often used to measure the ratio of information represented by principal components. In other words, it helps to identify the fraction of data variability contributed by a given set independent variable (features). In this study, we indent to explore if spike-representation of EEG signals provide additional information for improving the accuracy. Using PCA, we analyzed this metric on EEG signal & its spike-representation. As shown in Figure 5, it can be observed that for a given dataset from ERN, the spike-representation (red line) required more components than EEG signal (blue line) to explain 75% (black line) of variability in the data. This could imply that the proposed method was able to extract more unique independent features from the spike-representation of data and this could in turn enabled the classifier for better classification. Supplementary Figure 2 showcases a similar observation for P300 datasets.

#### 3.4.2. Correlation Analysis

**Figure 6:**
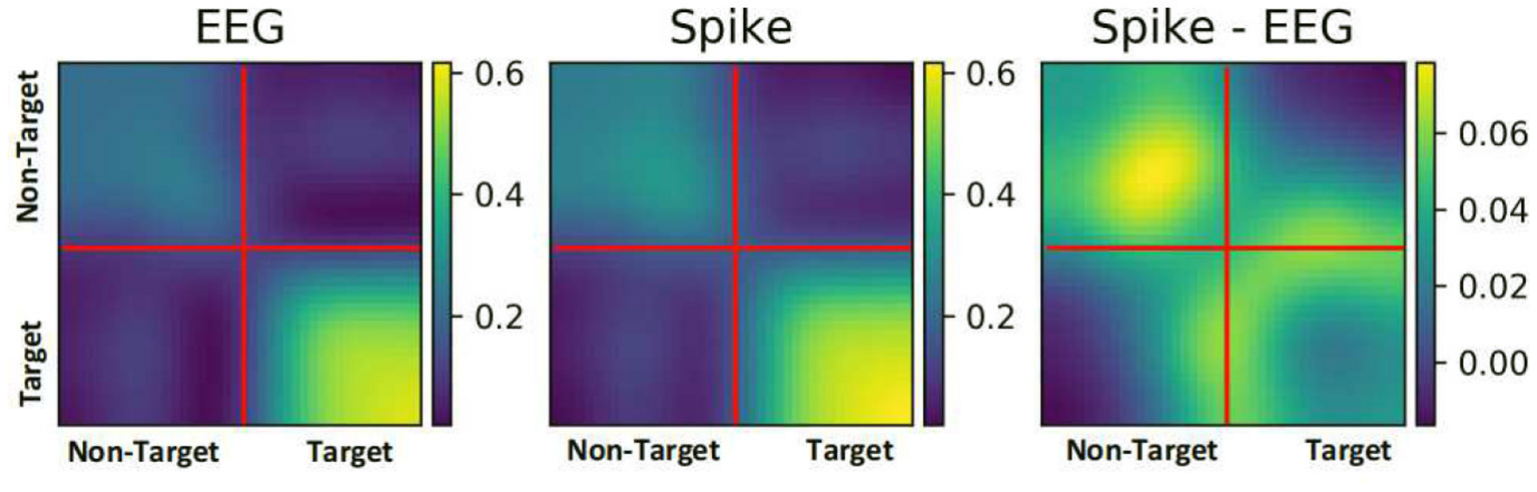
Pearson correlation among all the samples in an ERN dataset for both EEG signal and its spike-representation (from LIF). Spike representation forged a higher correlation among the samples of a given class.

To further understand the reason for performance enhancement, we analyzed the similarity among EEG samples and among their respective spike-representation. To this end, the correlation between all the samples of a given ERN dataset was estimated using Pearson's correlation technique. Figure 6, showcases the correlation matrix for all EEG samples, all spike-representation sample, and the difference of these two matrices (Spike-EEG plot). Each pixel represents the correlation between any two samples. It can be deduced from Spike-EEG plot that correlation among the spike-representation samples is slightly higher than of its EEG samples. This could imply that the proposed approach tends to enhance the similarity among the samples of given class (i.e., target and non-target classes) and this in-turn increased the performance. A similar increase in correlation for the P300 dataset is shown in Supplementary Figure 3.

### 3.5. Node Prioritization

For both ERN and P300, their prominent bio-maker (feature) can be found directly through their time-series signal. However, each subject's EEG template is unique and it is possible for the template to even contain noise and information irrelevant to the biomarker. Therefore, the number of nodes in RSNN required to generate an EEG template may vary depending on the complexity (i.e., variance) of the signal. As a result, we hypothesize that not all nodes in RSNN may contain reliable information, and considering only a few nodes' activities (referred to as node prioritization) could further improve the classification performance. The nodes with weights (from latent layer and output layer) that fall outside μ±1.5*σ range are assumed to be major contributors of EEG signals and as such only these nodes' activity is considered from spike-representation of EEG data. Here, μ and σ represent the mean and standard deviation of the weight distribution obtained after training the model. Also, it could be possible that such prioritization may not be ideal for all subjects. For this reason, we have compared to different modes: (a) node-prioritization and (b) threshold-based node prioritization. For the threshold-based approach, we have estimated the variance of the EEG template signal from each subject and considered the median value (from all variances) as a threshold for determining whether to apply node prioritization for a given subject dataset. In other words, node prioritization was applied to the subject data whose template signals have higher variance. As shown in Table 5, for ERN, the two mentioned prioritization modes improved the performance by a minor fraction compared to the performance compared to the original spike-representation performance. A similar observation was seen for P300 datasets as well (see Supplementary Table 1).

**Table 5:** Comparison of classification performance of spike-representation (from LIF) of ERN datasets with xGboost under different prioritization conditions such as: without node prioritization (WNP), node prioritization (NP), and threshold-based node prioritization (TNP).

**Dataset**	**WNP**	**NP**	**TNP**
S1	74.27	74.82	74.27
S2	82.00	83.67	82.00
S3	81.69	81.47	81.47
S4	89.51	90.10	90.10
S5	75.10	75.51	75.51
S6	73.33	73.67	73.33
S7	85.36	83.44	83.44
S8	74.23	69.76	69.76
S9	78.81	76.76	78.81
S10	89.61	88.41	89.61
S11	83.07	83.20	83.07
S12	80.67	84.33	84.33
S13	88.78	89.96	88.78
S14	87.80	90.84	90.84
S15	89.60	84.80	84.80
S16	60.67	81.19	81.19
S17	90.00	64.00	90.00
S18	79.60	90.00	90.00
S19	82.92	81.29	82.92
S20	80.83	84.00	80.83
**Avg**	81.39	81.56	**82.75**

*The bold values indicate the highest performance produced from all models*.

## 4. Discussion

EEG measurements had been a longstanding preferred mode of neuroimaging technique for BCI applications development due to its compactness, portability, and ease of use. However, EEG signals are often noisy and affect the performance of BCI systems. In addition, ERP-based BCI modalities such as ERN and P300 explicitly rely on the time-series segment of the signal for identifying the biomarkers. As such, they are often susceptible to noise and artifacts. Broadly, an EEG signal is the result of an accumulation of neural spiking information beneath the scalp surface. Theoretically, though the invasive recording is less prone to noise and contains robust information compared to EEG, it requires complex surgical effort. Extracting such predecessor spiking information responsible for an ERP could potentially help to understand the internal processes of BCI relevant activity and in turn, improve the BCI performance. Therefore, this study designed an approach based on SNN to generate relevant EEG signals (such as ERN and P300) and in turn extract its spike representation from the latent layer. This spike-representation is assessed toward BCI performance enhancements.

As shown in Section 3.2, the proposed approach was able to re-organize its spiking activity (see Figure 2B) and generate both ERN & P300 template signals. By evaluating using 5 × 10-fold CV metric with different standardized classifiers such as SVM, NVB, and xGboost, the spike-representation obtained from the LIF-based model has shown enhanced average BCI performance compared to baseline performance and further significantly for certain datasets. Also, besides LIF, the classification enhancement was observed with another spiking neuron, SNU. Besides ERN and P300, the model was also tested with non-ERP signals such as MI and we observed similar performance increment with spike-representation compared to (see Supplementary Table 2). In addition to classification, inferring the reason behind such performance improvement using PCA and correlation analysis has showcased that the proposed approach was able to extract more independent eigenvector (features) and increased within-class correlation and it facilitated the classifiers to better categorize the samples. To further validate the impact of SNN's trained weights, we have also compared the performance of transformed signal obtained with random weight, LSM and has shown that SNN's weights provided additional edge in performance enhancement. A current limitation of this study is that the threshold estimation requires information many datasets for node-prioritization. However, this technique offers advantages in terms of faster inference in edge devices as the prioritization enables node reduction profoundly. As a proof-of-concept, this also suggests that novel techniques such as pruning (Li et al., 2019) and deep-rewiring (Bellec et al., 2017) could be a desired approach for the future direction.

The template signal extraction was based on the assumption that a given stimulus should ideally produce similar neural activity over repeated trials (Mainen and Sejnowski, 1995). However, it could be plausible for a template containing irrelevant information to influence the spike representation too. Therefore, the future direction of this work includes the derivation of a clean template signal such that the representation of its in spike format can be more robust. Recent advances in deep learning have shown that *Representation learning* methods have enabled for better organization of data and in turn enhance the performance (Bengio et al., 2013; Le-Khac et al., 2020). For example, Khosla et al. (2020) introduced supervised contrastive learning where a loss function accounts for similarity and dissimilarity among samples for learning better representations. Adopting such techniques in the future direction could further enrich the spike representations.

## Data Availability Statement

The datasets used in this study are publicly available from https://osf.io/thsqg/, and https://zenodo.org/record/2649069/# (YPy2NDoRUUF).

## Author Contributions

SS and C-TL conceptualized the concept and methodology and wrote the manuscript. SS wrote the code and performed the simulations. All authors contributed to the article and approved the submitted version.

## Funding

This work was supported in part by the Australian Research Council (ARC) under discovery grant DP220100803 and DP210101093. Research was also sponsored in part by the Australia Defence Innovation Hub under Contract No. P18-650825, US Office of Naval Research Global under Cooperative Agreement Number ONRG - NICOP - N62909-19-1-2058, and AFOSR – DST Australian Autonomy Initiative.

## Conflict of Interest

The authors declare that the research was conducted in the absence of any commercial or financial relationships that could be construed as a potential conflict of interest.

## Publisher's Note

All claims expressed in this article are solely those of the authors and do not necessarily represent those of their affiliated organizations, or those of the publisher, the editors and the reviewers. Any product that may be evaluated in this article, or claim that may be made by its manufacturer, is not guaranteed or endorsed by the publisher.
