# Supplementary figures and images for: Spike-Representation of EEG Signals for Performance Enhancement of Brain-Computer Interfaces
Source: Front Neurosci. 2022 Apr 4;16:792318. doi: 10.3389/fnins.2022.792318 (PMC9014221; doi:10.3389/fnins.2022.792318)

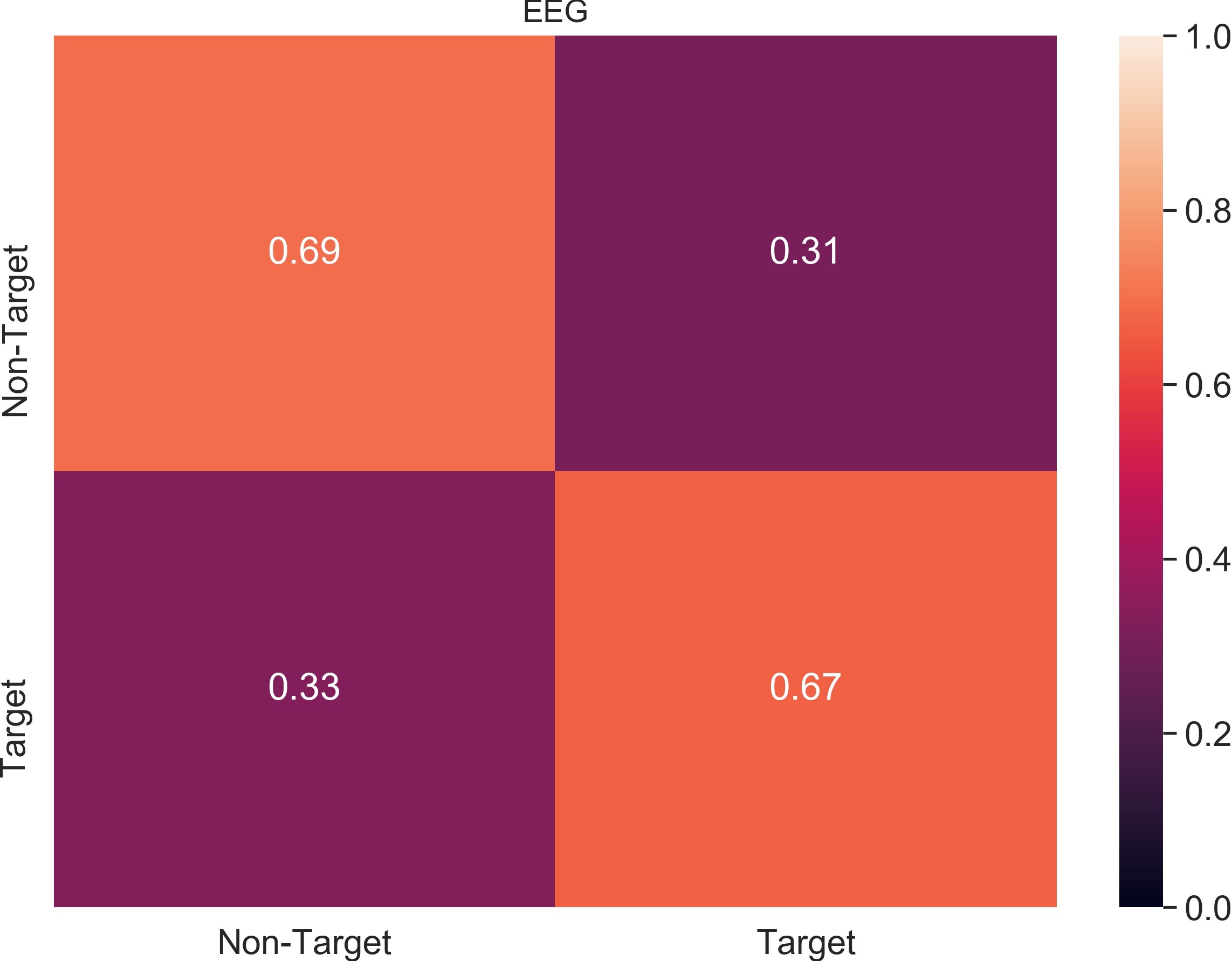

Supplement: Supplementary file 1 [file Image_1.jpg]

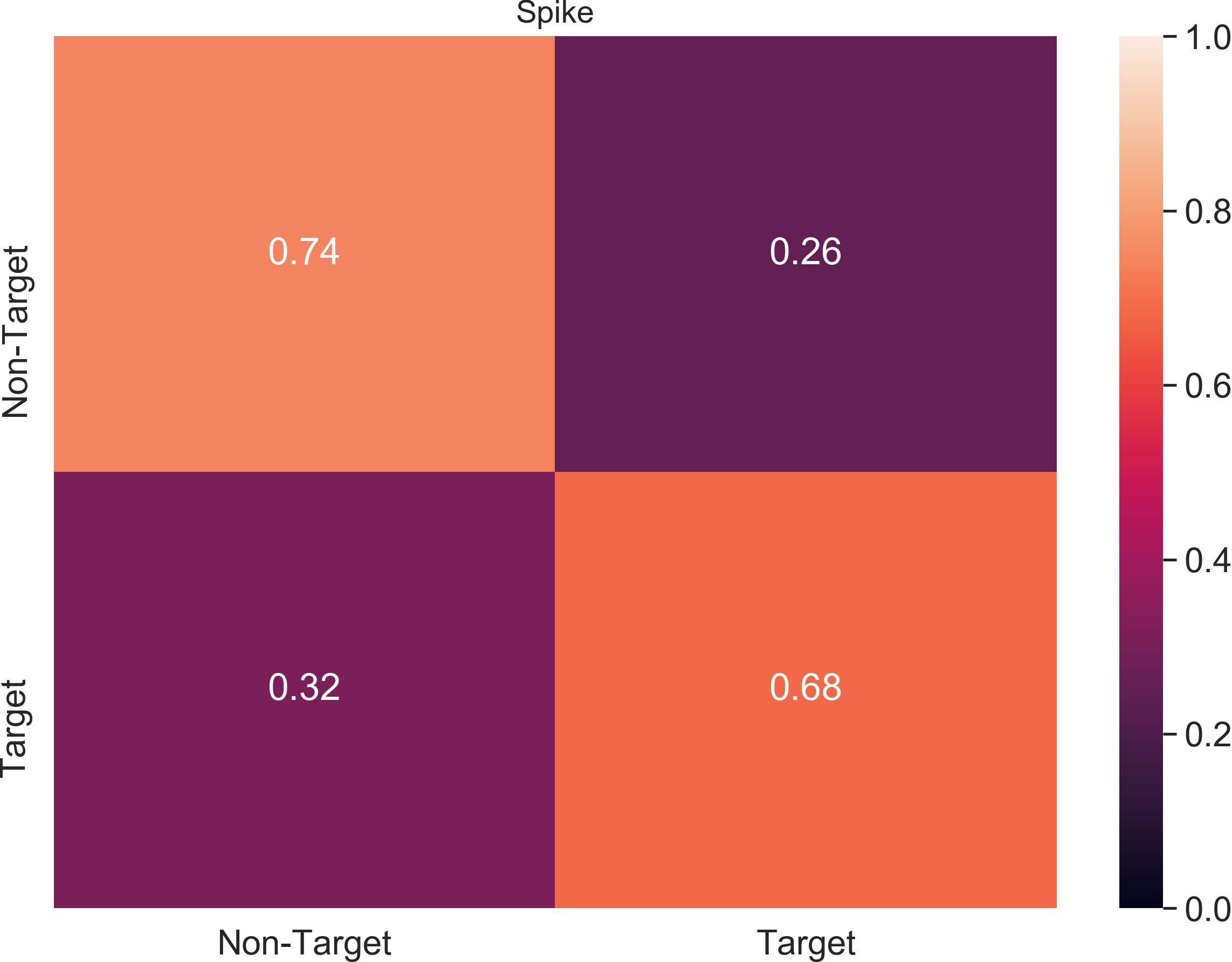

Supplement: Supplementary file 2 [file Image_2.jpg]
