# Supplementary material for: Spike-Representation of EEG Signals for Performance Enhancement of Brain-Computer Interfaces
Source: Front Neurosci. 2022 Apr 4;16:792318. doi: 10.3389/fnins.2022.792318 (PMC9014221; doi:10.3389/fnins.2022.792318)
Supplement: Supplementary file 3 [file Presentation_1.pdf]

Supplementary Material

1 P300 ANALYSIS

1.1 Confusion Matrix

Using xGboost classifier and 5x10 foldCV, the confusion matrix analysis for subject S3 that has displayed significant increase in performance for spike-representation is shown in Fig. S1.

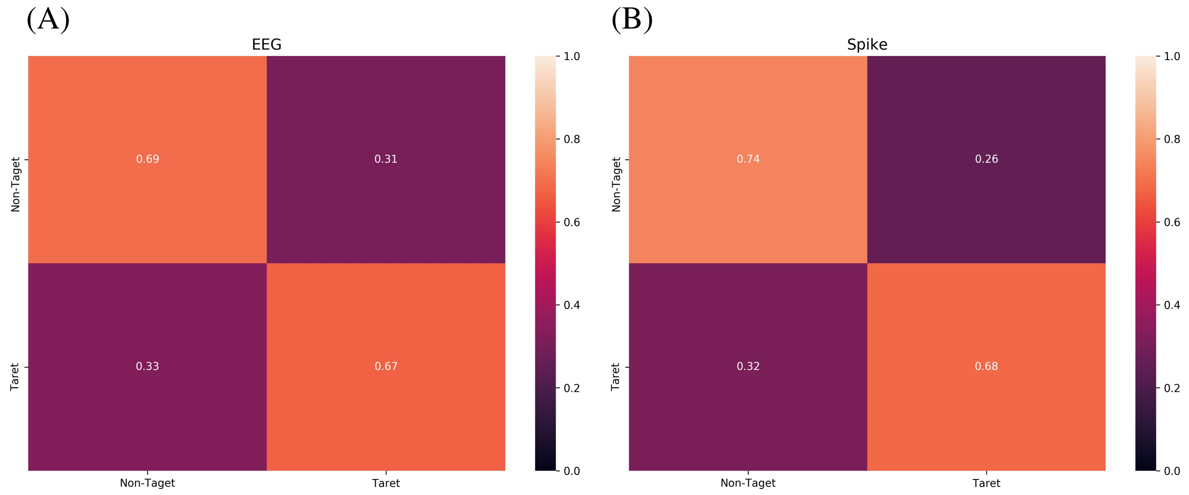

Figure S1. Confusion matrix analysis of **(A)** EEG and **(B)** Spike-representation (from LIF) of P300 dataset. Recognition of both *Target* and *Non-Target* categories improved for spike-representation compared to EEG signal.

1.2 Variance explanation

Similar to ERN, more independent eigenvectors (features) can be extracted from spike-representation signal to explain 75% variance in the data using PCA (see Fig. S2).

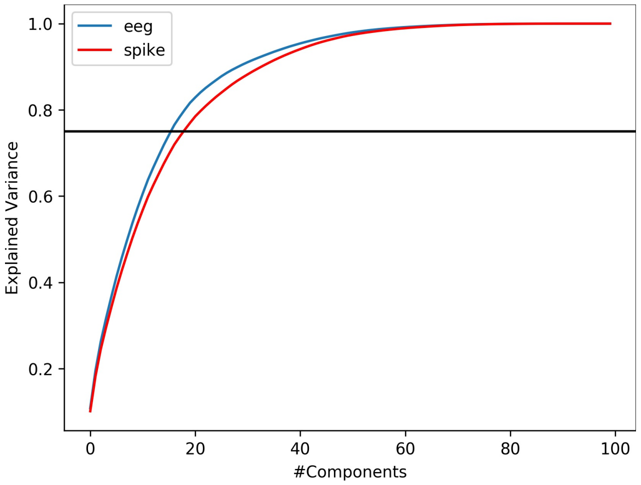

Figure S2. PCA based variance explanation of EEG signal (blue line) and its corresponding spike representation (red line) for P300 dataset (S13). Black line represents the 75% variance. Spike-representation (from LIF) was able to extract more independent eigenvector from EEG signal to account a given fraction of variance in the dataset.

1.3 Correlation analysis

For ERN we have observed increment in correlation among samples for both Target and Non-Target classes for spike-representation of the data. For P300, significant increase was observed for Target class samples as shown below in Fig. S3.

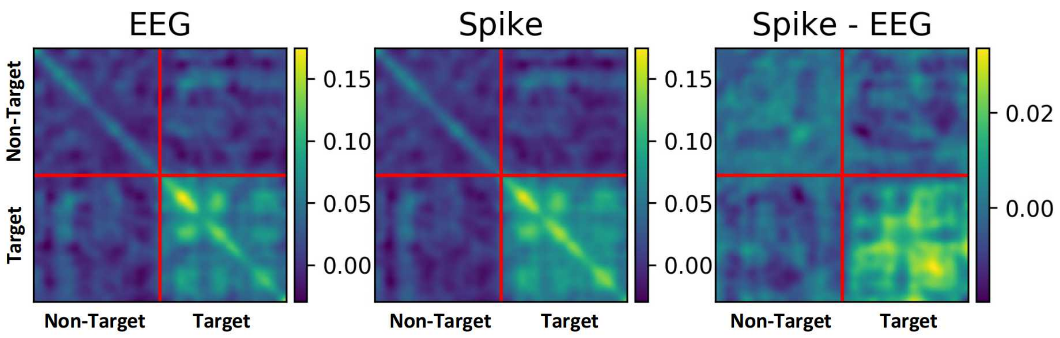

Figure S3. Pearson correlation among all the samples in a P300 dataset for both EEG signal and its spike-representation showcases that spike-representation produced higher correlation among the samples of Target class.

1.4 Node prioritization

Table S1. Comparision of classification performance of spike-representation of P300 datasets with xGboost under different prioritization conditions such as: without node prioritization (WNP), Node prioritization (NP) and Threshold-based node prioritization (TNP).

Dataset	WNP	NP	TNP
S1	71.61	71.84	71.84
S2	68.05	66.04	66.04
S3	71.37	71.93	71.93
S4	83.27	84.58	83.27
S5	65.04	65.00	65.04
S6	71.91	72.93	71.91
S7	66.19	66.98	66.98
S8	71.91	72.14	71.91
S9	71.89	73.01	71.89
S10	58.35	60.58	60.58
S11	66.41	66.69	66.69
S12	86.23	87.19	86.23
S13	62.66	63.90	62.66
S14	59.66	60.07	60.07
S15	62.71	64.38	62.71
S16	69.70	71.90	71.90
S17	74.06	72.50	74.06
S18	68.95	69.04	69.04
S19	73.70	72.85	72.85
S20	67.48	64.83	67.48
**Avg**	69.56	**69.92**	69.75

2 MI CLASSIFICATION

To further test of proposed approach on other BCI signals, we have used publicly available Motor Imagery (MI) datasets from BCI Competition IV. The data is processed to extract samples of two classes (Left hand MI and right hand MI) with 3 seconds duration and a sampling rate of 250Hz for . After processing, only C3 and C4 EEG channels were considered for SNN training as they are known to contain sufficient information for these two categories. Table S2 below showcases the classification performance of MI-EEG signals and their respective spike-representation using multi-layer perceptron (MLP).

Table S2. Comparison of classification performance of MI EEG signal and its spike-representation (with LIF) using MLP classifiers. For each subject, a 10 fold CV was applied where 90% of the samples were randomly selected for training and the remaining 10% for testing in each fold.

Dataset	EEG	LIF
S1	99.41	98.24
S2	59.41	63.53
S3	81.76	88.24
S4	48.75	61.86
S5	68.13	80.63
S6	60.63	57.50
S7	61.88	59.38
S8	65.00	65.83
S9	52.50	54.17
S10	53.75	56.25
S11	76.25	78.75
S12	63.33	63.33
S13	73.33	77.50
S14	60.50	62.50
S15	64.38	73.75
S16	65.00	68.13
**Avg.**	65.88	**69.35**
